# Supplementary material for: Liquid biopsy tracking during sequential chemo-radiotherapy identifies distinct prognostic phenotypes in nasopharyngeal carcinoma
Source: Nat Commun. 2019 Sep 2;10:3941. doi: 10.1038/s41467-019-11853-y (PMC6718666; doi:10.1038/s41467-019-11853-y)
Supplement: Supplementary file 1 — Supplementary Information [file 41467_2019_11853_MOESM1_ESM.pdf]

Supplementary Information

Liquid Biopsy Tracking during Sequential Chemo-radiotherapy Identifies Distinct Prognostic Phenotypes in Nasopharynx Cancer

Jiawei Lv et al.

SUPPLEMENTARY FIGURES

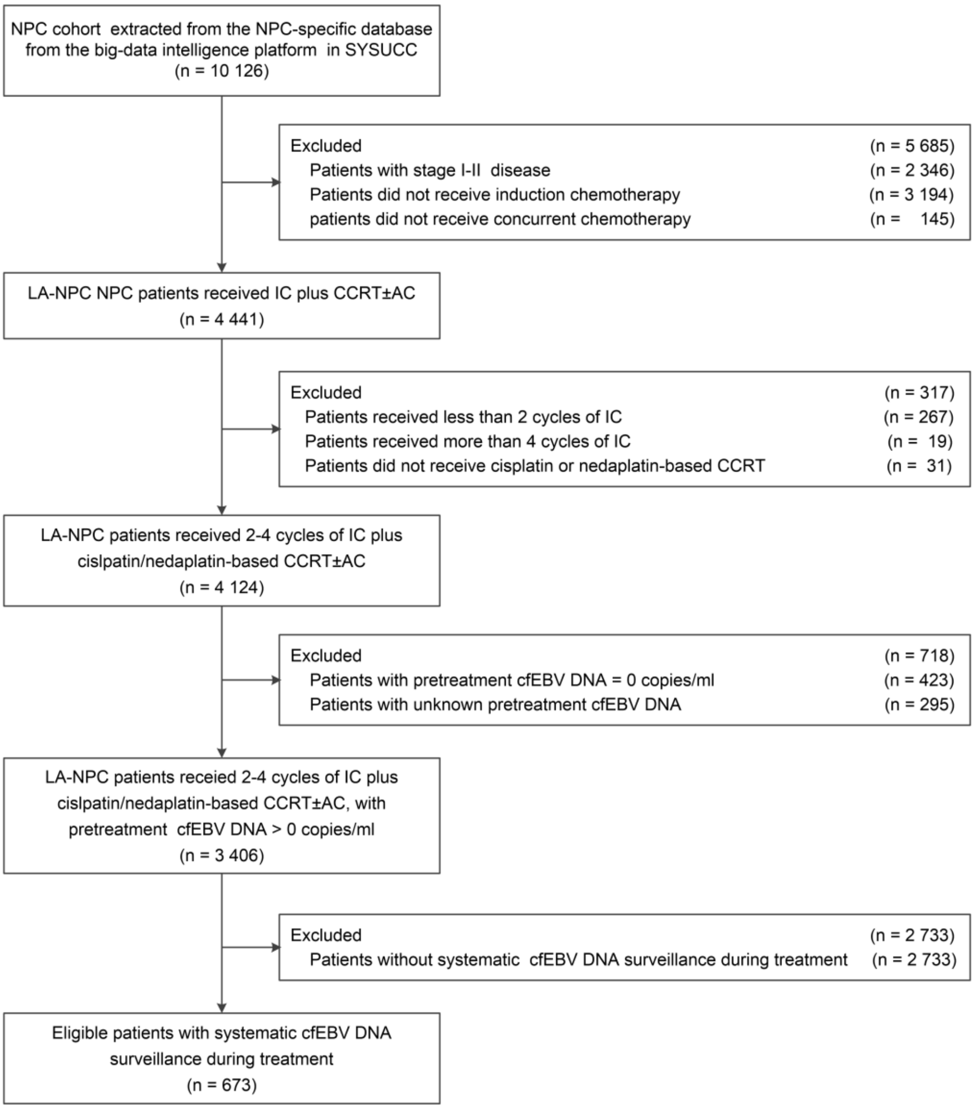

Supplementary Figure 1. Flowchart showing the study design and patient selection process. Medical records of 10,126 non-metastatic NPC patients were screened from an NPC-specific database within the Big-data intelligence framework, 673 LA-NPC patients who received IC+CCRT and had a detectable pre-treatment cfEBV DNA with longitudinal cfEBV DNA surveillance were selected stepwisely.

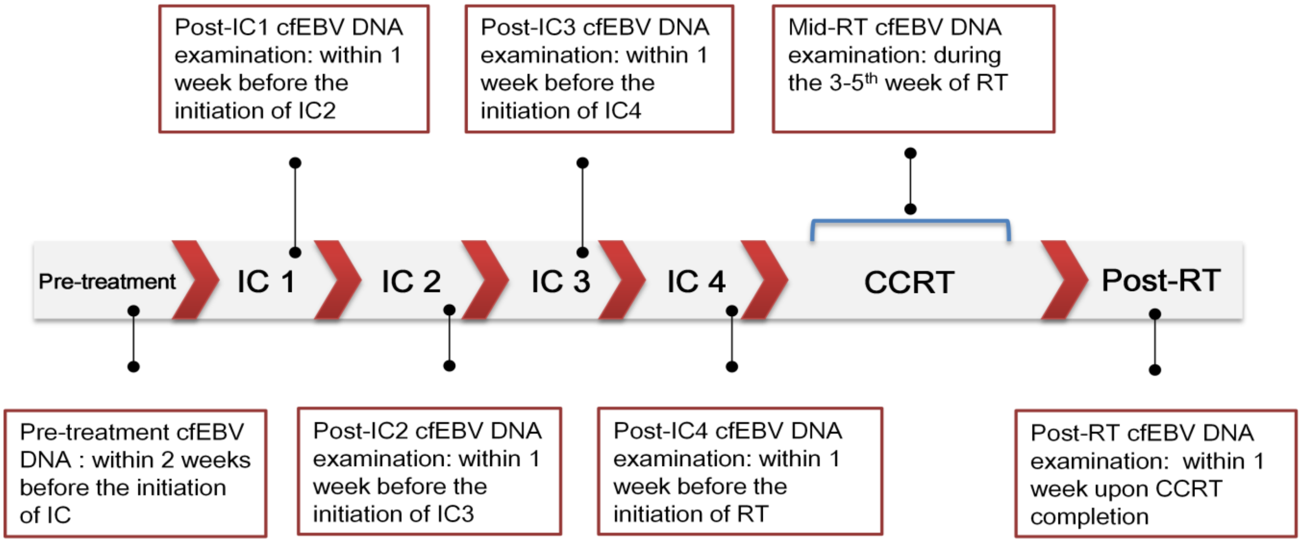

Supplementary Figure 2. Schema of liquid biopsy surveillance at different time-points during the treatment course of IC and CCRT.

cfEBV DNA was tested at the following time-points: two-week before IC initiation (pre-treatment); following every IC cycle (post-IC); during and within one-week upon CCRT completion (post-CCRT).

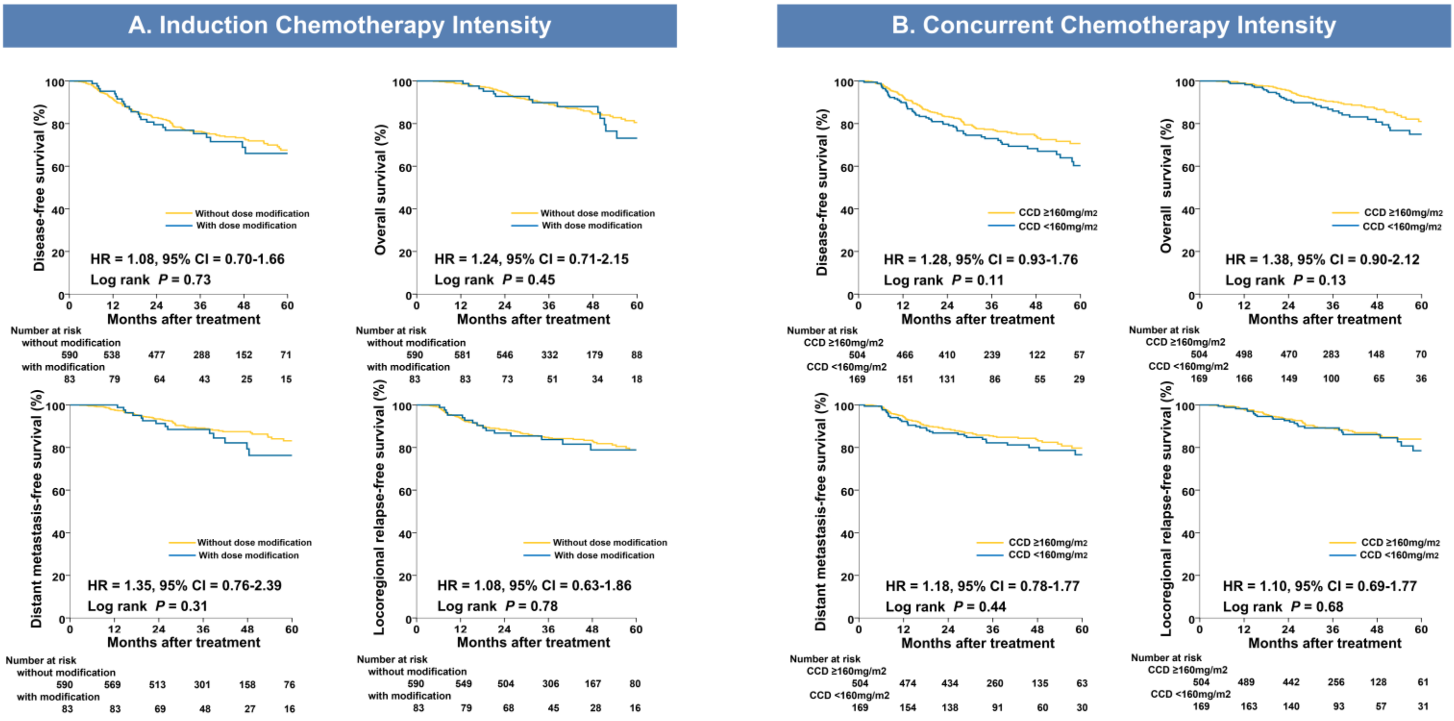

Supplementary Figure 3. **Kaplan-meier plots of survival outcomes for patients receiving different induction and concurrent chemotherapy intensities.**

(A). Disease-free survival (DFS), overall survival (OS), distant metastasis-free survival (DMFS) and locoregional relapse-free survival (LRFS) for patients with and without dose modification during induction chemotherapy; (B). DFS, OS, DMFS, and LRFS for patients receiving ≥160 mg/m^2 and <160 mg/m^2 concurrent cisplatin dose.

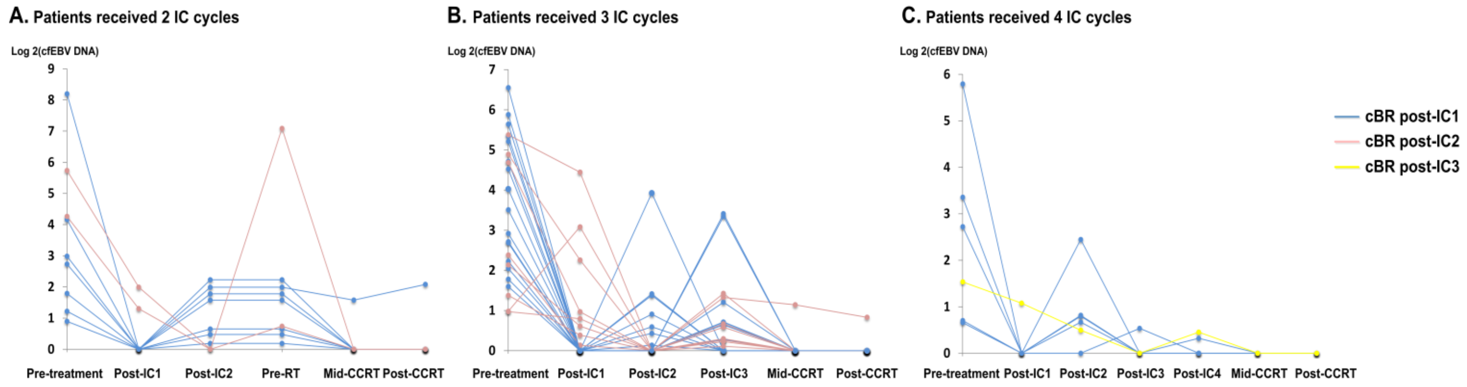

Supplementary Figure 4. The longitudinal cfEBV DNA response in patients with early DNA bounce.

(A). cfEBV DNA bounce for patients receiving 2 cycles of induction chemotherapy (IC); (B). cfEBV DNA bounce for patients receiving 3 cycles of IC; (C). cfEBV DNA bounce for patients receiving 4 cycles of IC.

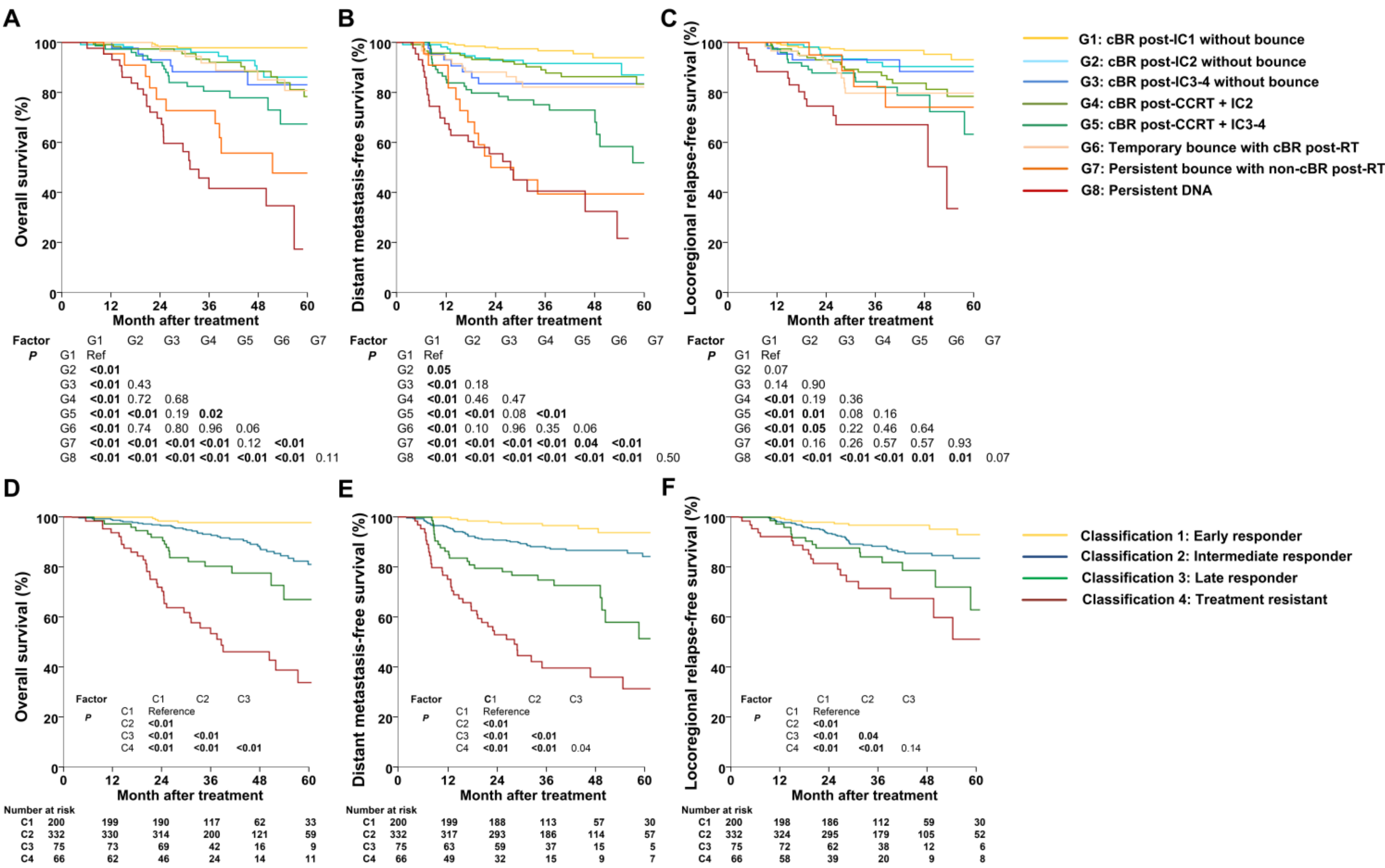

Supplementary Figure 5. Kaplan-meier plots of survival outcomes for the eight cfEBV DNA response subgroups and the four cfEBV DNA phenotypic clusters.

(A). Overall survival (OS) for the eight cfEBV DNA response subgroups; (B). distant metastasis-free survival (DMFS) for the eight cfEBV DNA response subgroups; (C). locoregional relapse-free survival (LRFS) for the eight cfEBV DNA response subgroups; (D). OS for the four cfEBV DNA phenotypic clusters; (E). DMFS for the four cfEBV DNA phenotypic clusters, and (F). LRFS for the four cfEBV DNA phenotypic clusters.

SUPPLEMENTARY TABLES

Supplementary Table 1. Basic characteristics of cohort of 673 locoregionally advanced nasopharyngeal carcinoma patients

Characteristics	No.(%)
**Sex**	
Male	497 (73.8)
Female	176 (26.2)
**Age (years)**	
<45	373 (55.4)
45–65	279 (41.5)
>66	21 (3.1)
**Year of diagnosis**	
2010–2013	285 (42.3)
2014–2015	388 (57.7)
**WHO pathological type**	
Undifferentiated non-keratinizing	659 (97.9)
Differentiated non-keratinizing	14 (2.1)
**T-category**[*]	
T1	32 (4.8)
T2	66 (9.8)
T3	330 (49.0)
T4	245 (36.4)
**N-category**[*]	
N0	30 (4.5)
N1	241 (35.8)
N2	211 (31.4)
N3	191 (28.4)
**TNM**[†]	
III	283 (42.1)
IV	390 (57.9)
**Pretreatment cfEBV DNA (copies/ml)**	
≤ 2 000	138 (20.5)
2 000–20 000	252 (37.4)
> 20 000	283 (42.1)
**Treatment**	
IC + CCRT	631 (93.8)
IC + CCRT + AC	42 (6.2)

**IC cycles**	
2 cycles	353 (52.5)
3 cycles	268 (39.8)
4 cycles	52 (7.7)
**IC regimens**	
TPF	387 (57.5)
TP	176 (26.2)
PF	58 (8.6)
GP	45 (6.7)
Others†	7 (1.0)
**CCRT regimens**	
Cisplatin	591 (87.8)
Nedaplatin	82 (12.2)
**AC regimens**	
GP	2 (4.8)
Tegafur-uracil	23 (54.8)
Capecitabine	17 (40.4)

Abbreviations: AC = adjuvant chemotherapy; CCRT = concurrent chemotherapy; IC = induction chemotherapy; EBV DNA = Epstein–Barr virus deoxyribonucleic acid; GP = gemcitabine and cisplatin; PF = cisplatin and 5-fluorouracil; T = tumor; TP = docetaxel and cisplatin; TPF = docetaxel, cisplatin, and 5-fluorouracil; N = node

* According to the 8th edition of the AJCC/UICC staging system.
† Others include patients who underwent regimen alterations during induction chemotherapy, such as those who received two cycles of TPF plus one cycle of TP.

Supplementary Table 2. The distributions of T- and N category in the eight cfEBV DNA response groups and the four cfEBV DNA phenotypic clusters

cfEBV DNA Response	T-categories				P^*-value	N-categories				P^*-value
	T1 (N,%)	T2 (N,%)	T3 (N,%)	T4 (N,%)		N0 (N,%)	N1 (N,%)	N2 (N,%)	N3 (N,%)	
**cfEBV DNA Response Groups**					0.15					**< 0.01**
**Group 1**	12 (37.5)	9 (13.6)	104 (31.5)	75 (30.6)		12 (40.0)	86 (35.7)	61 (28.9)	41 (21.5)	
**Group 2**	5 (15.6)	10 (15.2)	59 (17.9)	39 (15.9)		4 (13.3)	41 (17.0)	35 (16.6)	33 (17.3)	
**Group 3**	3 (9.4)	3 (4.5)	23 (7.0)	14 (5.7)		2 (6.7)	14 (5.8)	15 (7.1)	12 (6.3)	
**Group 4**	5 (15.6)	17 (25.8)	60 (18.2)	35 (14.3)		2 (6.7)	40 (16.6)	43 (20.4)	32 (16.8)	
**Group 5**	2 (6.3)	10 (15.2)	27 (8.2)	36 (14.7)		3 (10.0)	21 (8.7)	20 (9.5)	31 (16.2)	
**Group 6**	1 (3.1)	8 (12.0)	30 (9.1)	20 (8.2)		7 (23.3)	23 (9.5)	14 (6.6)	15 (7.9)	
**Group 7**	2 (6.3)	1 (1.5)	9 (2.7)	10 (4.1)		0 (0.0)	8 (3.3)	8 (3.8)	6 (3.1)	
**Group 8**	2 (6.3)	8 (12.1)	18 (5.5)	16 (6.5)		0 (0.0)	8 (3.3)	15 (7.1)	21 (11.0)	
**cfEBV DNA Phenotypic Clusters**					**0.02**					**< 0.01**
**Cluster 1**	12 (37.5)	9 (13.6)	104 (31.5)	75 (30.6)		12 (40.0)	86 (35.7)	61 (28.9)	41 (21.5)	
**Cluster 2**	14 (43.8)	28 (57.6)	172 (52.1)	108 (44.1)		15 (50.0)	18 (49.0)	107 (50.7)	92 (48.2)	
**Cluster 3**	2 (6.3)	10 (15.2)	27 (8.2)	36 (14.7)		3 (10.0)	21 (8.7)	20 (9.5)	31 (16.2)	
**Cluster 4**	4 (12.5)	9 (13.6)	27 (8.2)	26 (10.6)		0 (0.0)	16 (6.6)	15 (10.9)	21 (14.1)	

Abbreviations: EBV DNA = Epstein–Barr virus deoxyribonucleic acid; T= tumor; N = node
* *P*-values were calculated by fisher exact test

Supplementary Table 3. Radiotherapy treatment details

Treatment Interruption	No of patients (%)
**Reason for Interruption**	
Myelosuppression	7
Radiation-related adverse events*	3
Atrial fibrillation	1
Stomachache	1
**Time of Interruption (Days)**	
< 3	4
3-7	6
> 7	2
**cfEBV DNA Response Phenotypes**	
Cluster 1	4
Cluster 2	6
Cluster 3	1
Cluster 4	1

* included radiation-induced mucositis and dermatitis.

Supplementary Table 4. Comparison of treatment-related confounders between the different cfEBV DNA response phenotypes

Therapeutic confounders	Cluster 1	Cluster 2	Cluster 3	Cluster 4	*P*-value*
**Wait time**					0.98
≤ 1 week	186 (93.0)	308 (92.8)	70 (93.3)	62 (93.9)	
> 1week	14 (7.0)	24 (7.2)	5 (6.7)	4 (6.1)	
**Treatment Interruptions**					0.97
No	196 (98.0)	326 (98.2)	74 (98.7)	65 (98.5)	
Yes	4 (2.0)	6 (1.8)	1 (1.3)	1 (1.5)	
**IC regimens**					0.28
Triplets	125 (62.5)	186 (56.0)	43 (57.3)	33 (50.0)	
Doublets	75 (37.5)	146 (44.0)	32 (43.7)	33 (50.0)	
**CCD**					0.79
≥ 160 mg/m^2	49 (24.5)	82 (24.7)	18 (24.0)	46 (30.0)	
<160 mg/m^2	151 (75.5)	250 (75.3)	57 (76.0)	20 (69.7)	

Abbreviations: CCD = cumulative concurrent chemotherapy dose; IC = induction chemotherapy

* Two-sided *P*-values were calculated using the Chi-square test

Supplementary Table 5. The number of IC cycles between patients with cBR and non-cBR post-IC_{1-2}

IC cycles	Post-IC_1 cfEBV DNA		*P*-value*	Post-IC_2 cfEBV DNA		*P*-value*
	**cBR**	**Non-cBR**		**cBR**	**Non-cBR**	
2 IC cycles	132 (53.9)	221 (51.6)		206 (56.3)	147 (47.9)	
3 IC cycles	88 (35.9)	180 (42.1)	0.10	134 (36.6)	135 (44.0)	0.09
4 IC cycles	25 (10.2)	27 (7.7)		26 (7.1)	25 (8.1)	

Abbreviations: IC = induction chemotherapy; cBR = complete biological response; cfEBV DNA = Epstein–Barr virus deoxyribonucleic acid.
* Two-sided *P*-values were calculated using the Chi-square test

Supplementary Table 6. Cox proportional hazards analyses of clinical-relevant covariates in 673 locoregionally advanced nasopharyngeal carcinoma patients

Endpoint	Variable	HR	95% CI	*P*-value*
**DFS**	Pre-treatment cfEBV DNA (>2 000 *vs.* ≤2 000 copies/ml)	1.51	0.91–2.50	0.11
	IC regimens (triplets *vs*. doublets)	1.01	0.70–1.30	0.95
	CCD (≥160mg/m^2 *vs*. <160mg/m^2)	1.35	0.97–1.89	0.08
	T-category			0.88
	T3 *vs.* T1-2 (reference)	1.08	0.69–1.69	
	T4 *vs.* T1-2 (reference)	1.13	0.70–1.81	
	N-category			**0.02**
	N2 *vs.* N0-1(reference)	1.69	1.16–2.48	
	N3 *vs.* N0-1(reference)	1.49	1.04–2.24	
**OS**	Pre-treatment cfEBV DNA (>2 000 *vs.* ≤2 000 copies/ml)	1.23	0.60–2.51	0.58
	IC regimens (triplets *vs*. doublets)	0.94	0.85–1.43	0.16
	CCD (≥160mg/m^2 *vs*. <160mg/m^2)	1.48	0.95–2.31	0.08
	T-category			0.95
	T3 *vs.* T1-2 (reference)	1.04	0.67–1.62	
	T4 *vs.* T1-2 (reference)	1.10	0.69–1.78	
	N-category			**0.03**
	N2 *vs.* N0-1(reference)	1.58	0.91–2.77	
	N3 *vs.* N0-1(reference)	1.87	1.06–3.29	
**DMFS**	Pre-treatment cfEBV DNA (>2 000 *vs.* ≤2 000 copies/ml)	1.54	0.74–2.81	0.12
	IC regimens (triplets *vs*. doublets)	0.70	0.47–1.04	0.08
	CCD (≥160mg/m^2 *vs*. <160mg/m^2)	1.26	0.82–1.94	0.28

	T-category			0.60
	T3 *vs.* T1-2 (reference)	0.92	0.56–1.52	
	T4 *vs.* T1-2 (reference)	0.77	0.44–1.34	
	N-category			**0.01**
	N2 *vs.* N0-1(reference)	1.61	1.06–2.75	
	N3 *vs.* N0-1(reference)	2.17	1.27–3.69	
**LRFS**	Pre-treatment cfEBV DNA (>2 000 *vs.* ≤2 000 copies/ml)	1.13	0.65–2.32	0.52
	IC regimens (triplets *vs*. doublets)	1.31	0.85–2.03	0.24
	CCD (≥160mg/m^2 *vs*. <160mg/m^2)	1.11	0.68–1.79	0.69
	T-category			**0.03**
	T3 *vs.* T1-2 (reference)	3.57	1.26–10.13	
	T4 *vs.* T1-2 (reference)	3.92	1.36–11.32	
	N-category			0.13
	N2 *vs.* N0-1(reference)	1.45	0.88–2.39	
	N3 *vs.* N0-1(reference)	0.82	0.45–1.50	

Abbreviations: AHR = adjusted hazard ratio; CI = confidence interval; DFS = disease-free survival; DMFS = distant metastasis-free survival; LRFS = locoregional relapse-free survival; EBV DNA = Epstein–Barr virus deoxyribonucleic acid; OS = overall survival

* Pre-treatment cfEBV DNA(>2 000 *vs*. ≤2 000copies/ml), T-category (T4 *vs*. T3 *vs*. T1-2), N-category (N3 *vs*. N2 *vs*. N0-1), IC regimens (triplets *vs*. doublets), cumulative concurrent chemotherapy dose (≥160mg/m^2 *vs*. <160mg/m^2), and four cfEBV DNA response clusters were included in the Cox regression model

Supplementary Table 7. Baseline characteristics of patients receiving different IC intensities among intermediate and late responders with or without cBR post-IC_2

**Characteristics**[*]	**cBR post-IC_2**		***P*-value**[†]	**Non-cBR post-IC_2**		***P*-value**[†]
	**2 IC cycles**	**3-4 IC cycles**		**2 IC cycles**	**3-4 IC cycles**	
**Sex**			0.08			0.47
Male	54 (79.4)	31 (68.9)		92 (74.2)	103 (78.0)	
Female	13 (19.4)	14 (31.1)		32 (25.8)	25 (22.0)	
**Age (years)**			0.42			0.54
<45	32 (47.8)	25 (55.6)		61 (49.2)	70 (53.0)	
≥45	35 (52.2)	20 (44.4)		63 (50.8)	62 (47.0)	
**Year of diagnosis**			0.61			0.15
2010–2013	28 (41.8)	21 (46.7)		58 (46.8)	50 (37.9)	
2014–2015	39 (58.2)	24 (53.3)		66 (53.2)	82 (62.1)	
**T-category**[‡]			0.22			0.15
T1-2	10 (14.9)	5 (11.1)		22 (17.7)	19 (14.4)	
T3	38 (56.7)	20 (44.4)		64 (51.6)	57 (43.2)	
T4	19 (28.4)	20 (44.4)		38 (30.6)	56 (42.4)	
**N-category**[‡]			0.99			0.24
N0–1	26 (38.8)	18 (40.0)		48 (38.7)	44 (33.3)	
N2	21 (31.3)	14 (31.1)		44 (35.5)	41 (31.1)	
N3	20 (29.9)	13 (28.9)		32 (25.8)	47 (35.6)	
**TNM**[†]			0.15			0.11
III	33 (49.3)	16 (35.6)		57 (46.0)	48 (36.4)	

IV	34 (50.7)	29 (64.4)		67 (54.0)	84 (63.6)	
**Pretreatment cfEBV DNA**			0.94			0.09
≤ 2 000	16 (23.9)	12 (26.7)		14 (11.3)	20 (15.2)	
2 000–20 000	19 (28.4)	12 (26.7)		55 (44.4)	44 (33.1)	
> 20 000	32 (47.8)	21 (46.6)		55 (44.4)	68 (51.5)	
**IC regimens**§			0.27			0.11
Triplets	37 (55.2)	27 (60.0)		66 (48.4)	77 (58.3)	
Doublets	30 (44.8)	18 (40.0)		64 (51.6)	55 (41.7)	
**CCD**			0.88			0.46
≥ 160 mg/m^2	51 (75.0)	33 (73.3)		97 (78.2)	98 (74.2)	
<160 mg/m^2	17 (25.0)	12 (26.7)		27 (22.8)	34 (25.8)	

Abbreviations: CCD = cumulative concurrent chemotherapy dose; IC = induction chemotherapy; cfEBV DNA = Epstein–Barr virus deoxyribonucleic acid; T = tumor; N = node.

* Patients with IC regimens alteration were excluded from the analysis
†Two-sided *P*-values were calculated using the Chi-square test
‡ According to the 8th edition of the American Joint Committee on Cancer stage system
§Triplets refers to docetaxel/cisplatin/fluorouracil (TPF); doublets includs docetaxel/cisplatin (TP), cisplatin/fluorouracil (PF), gemcitabine plus cisplatin (GP).

SUPPLEMENTARY MATERIALS AND METHODS

Data extraction and study population

The NPC-specific database from the big-data intelligence platform at Sun Yat-Sen University Cancer Centre (SYSUCC), China was established in 2015. It allows oncologists to set query conditions in a flexible manner, quickly search for eligible cases, clearly visualize data, and enroll eligible cases into cohort or prospective studies. Furthermore, real-time medical records of patients is organized, integrated, and updated automatically from a number of clinical business systems based on a well-designed data model and algorithm in this database. Detailed description of this big-data platform was stated in our previously published work[1].

The NPC-specific database from the big-data intelligence platform in SYSUCC was used to identify a cohort of 10,126 patients with histological-proven, non-disseminated NPC, diagnosed between April 2009 and Dec 2015. Patient demographic characteristics, diagnostic and therapeutic information were accurately collected by using the search terms "diagnosis," "histology type," "age at first diagnosis," "sex," "disease stage," "radiotherapy technology," "chemotherapy regimens," "EBV DNA".

Diagnosis, stage and follow-up

All patients underwent pretreatment evaluations, including physical examination, hematology and biochemistry profiling, EBV DNA testing, fiberoptic nasopharyngoscopy, magnetic resonance imaging (MRI) of the suprasellar cistern to the collarbone, computed tomography (CT), abdominal ultrasonography, whole-body bone scan (ECT), or 18F-fluorodeoxyglucose positron emission tomography–computed tomography (PET-CT).

Restaging was performed by two experienced radiation oncologists specializing in head and neck cancer according to the 8th edition of the American Joint Commission on Cancer (AJCC) staging system[2]; disagreements were resolved by consensus.

Patients were followed-up every 3 months during the first 2 years and every 6 months for 3 years thereafter. During the visits, clinical examinations, nasopharyngoscopy, and cfEBV DNA examinations were routinely performed.

Patients in whom recurrence or metastasis was clinically suspected were recommended to undergo magnetic resonance imaging (MRI), abdominal sonography, whole-body bone scanning (ECT), or 18F-fluorodeoxyglucose positron emission tomography–computed tomography (PET-CT), followed by confirmatory cytological biopsies if possible.

Treatment protocol and longitudinal cfEBV DNA surveillance

Target volumes were delineated according to the institutional guidelines, which complies with *the International Commission on Radiation Units and Measurements reports 50 and 62*[3, 4]. The prescribed doses were 66–72 Gy/28–33 fractions to the planning target volume (PTV) of the primary gross tumor volume (GTVnx), 64–70 Gy/28–33 fractions to the PTV of the GTV of the involved lymph nodes (GTVnd), 60–63 Gy/28–33 fractions to the PTV of the high-risk clinical target volume (CTV1), and 54–56 Gy/28–33 fractions to the PTV of the low-risk CTV (CTV2). All patients were treated following a routine schedule of one fraction daily 5 days per week for a total of 6–7 weeks.

The IC regimens comprised of two to four cycles of 3-weekly TPF, TP, PF, and GP. The TPF regimen consisted of docetaxel 60 mg/m^2 on day 1, cisplatin 60 mg/m^2 on day 1, and 5-FU 500 mg/m^2/d continuously from day 1 to day 5. The TP regimen consisted of docetaxel 75 mg/m^2 on day 1 and cisplatin 75 mg/m^2 on day 1. The PF regimen comprised cisplatin 75 mg/m^2 on day 1 and 5-FU 500 mg/m^2/d continuously from day 1 to day 5. The GP regimen comprised cisplatin 80 mg/m^2 on day 1 and gemcitabine 1 000 mg/m^2 on day 1 and day 8. The regimens were repeated every three weeks for 2–4 cycles.

The concurrent chemotherapy consisted of 30–40 mg/m^2 cisplatin/nedaplatin administered every week for a maximum of seven cycles, 80 mg/m^2 cisplatin/nedaplatin administered every 3 weeks for a maximum of three cycles, or 100 mg/m^2 cisplatin/nedaplatin administered every 3 weeks for a maximum of three cycles, beginning on the first day of RT.

Dose modifications for haematological and nonhaematologicaltoxicity during IC and CCRT were based on the nadir blood counts and interim toxicities of the preceding cycle[5, 6]. Reductions in the dose of docetaxel (T) were planned for neutropenia, thrombocytopenia, impaired liver function, severe diarrhoea, or mucositis. Docetaxel dose had to be reduced by one level

(10 mg/m²) if the patient had a second episode of febrile neutropenia, neutropenic infection, neutropenia lasting for longer than 7 days, first episode of grade 4 thrombocytopenia, aspartate aminotransferase, alanine aminotransferase, or alkaline phosphatase (more than 2.5 to 5.0 times the upper limit of normal), first episode of grade 4 diarrhoea, or second episode of grade 3 diarrhoea, or grade 4 mucositis. Modifications in the dose of cisplatin (DDP) were planned for neutropenia, thrombocytopenia, nephrotoxicity, or neurotoxicity. Cisplatin dose had to be reduced by one level (10 mg/m^2 in induction phase and 20 mg/m^2 in concurrent phase) if the patient had grade 3 neutropenia or grade 2 thrombocytopenia (concurrent phase only), creatinine clearance of 40–60 mL/min, or grade 2 neurotoxicity. Modifications in the dose of fluorouracil (5FU) were made for diarrhoea or mucositis. Fluorouracil dose had to be reduced by one level (100 mg/m^2) if the patient had their first episode of grade 3–4 diarrhoea or grade 3 mucositis. Modifications in the dose of gemcitabine (G) were planned for neutropenia, thrombocytopenia, impaired liver function, or severe mucositis. Gemcitabine dose had to be reduced by one level (200 mg/m^2) if the patient had first episode of grade 4 neutropenia, febrile neutropenia, neutropenic infection, first episode of grade 3 thrombocytopenia, aspartate aminotransferase, alanine aminotransferase, alkaline phosphataseor bilirubin (more than 1.0 to 2.0 times the upper limit of normal), or first episode of grade 2 pulmonary toxicity. Chemotherapy was stopped completely if the patient had creatinine clearance of less than 40 mL/min; aspartate aminotransferase, alanine aminotransferase, or alkaline phosphatase more than five times the upper limit of normal; second episode of grade 4 diarrhoea; or grade 3 or higher neurotoxicity or ototoxicity.

cfEBV DNA was tested at the following time-points: two-week before IC initiation (pre-treatment); following every IC cycle (post-IC); during and within one-week upon CCRT completion (post-CCRT). Peripheral blood (3 ml) were obtained at each time-point in an EDTA tube and centrifuged at 1600 x g for 15 min for isolation of plasma and PBC. Viral DNA was extracted using the QIAamp Blood Kit (Qiagen, Hilden, Germany) and stored at -80°C until further processing. A total of 500 μl plasma samples were used for DNA extraction per column and a final elution volume of 50 μl was used to elute the DNA from the extraction column. A real-time quantitative polymerase chain reaction (PCR)

system was developed for plasma EBV DNA detection toward the BamHI-W region of the EBV genome[7]. The sequences of the forward and reverse primers were: 5`-GCCAG AGGTA AGTGG ACTTT-3` and 5`-TACCA CCTCC TCTTC TTGCT-3` respectively. A dual fluorescently-labelled oligomer, 5`-(FAM) CACAC CCAGG CACAC ACTAC ACAT (TAMRA)-3` served as the probe. Amplifications were performed in a Applied Biosystems 7700 Sequence Detector and then analyzed using the Sequence Detection System software (version 1.6.3) developed by Applied Biosystems (Foster City, CA). The plasma EBV DNA concentration was calculated using the following equation: $C = Q \times (VDNA/VPCR) \times (1/VEXT)$, in which C represents the target concentration in plasma (copies/ml), Q represents the target quantity (copy number) determined by PCR, VDNA represents the total volume of DNA obtained after extraction (typically 50 μl/Qiagen extraction), VPCR represents the volume of DNA solution used for PCR (typically 2 μl) and VEXT represents the volume of plasma extracted (typically 0.5 ml).

Study endpoints and statistical analysis

Primary endpoint was disease-free survival (DFS), which was defined as the time from treatment initiation to tumor progression or death. The secondary endpoint was overall survival (OS), which was defined as the time from the date of treatment initiation to death of any cause; distant metastasis-free survival (DMFS) was defined as the time to tumor metastasis; and locoregional relapse-free survival (LRFS) was defined as the time to the first locoregional relapse.

The basic characteristics among groups were compared using the $\chi 2$-test (Fisher's exact test or Pearson's $\chi 2$-test where appropriate) for categorical variables, and the Mann-Whitney U test or Kruskal-Wallis test for continuous variables. Kaplan-Meier survival analysis was used to estimate the actuarial survival rates; log-rank tests were used for comparisons. Univariable and multivariable tests of association between the cBR phenotypes, clinical and treatment parameters (including pre-treatment cfEBV DNA, T- and N-categories, IC regimens, and cumulative concurrent chemotherapy dose), and survival were performed using Cox regression. For the cBR phenotypes,

we performed a supervised clustering based on relative intergroup HR_{DFS} to reduce the number of subgroups. The proportional hazards assumption was verified using time-dependent Cox regression analysis[8]. Landmark analysis was also performed, which started the survival clock for patients at the time-point they were placed into each classified group[8], in order to further control the biases of physicians decision-making that may be influenced by cfEBV DNA results during treatment. All statistical analyses were performed with R version 3.4.4 (http://www.r-project.org) and SPSS version 23.0 (SPSS Inc., Chicago, IL, USA). Statistical significance was set at two-sided P <0.05. The clustering plot was done using the ggplot2 package on *R*.

SUPPLEMENTARY REFERENCE

1. Lv JW, Chen YP, Huang XD, Zhou GQ, Chen L, Li WF*, et al.* Hepatitis B virus screening and reactivation and management of patients with nasopharyngeal carcinoma: A large-scale, big-data intelligence platform-based analysis from an endemic area. *Cancer* 2017, **123**(18)**:** 3540-3549.

2. Colevas AD, Yom SS, Pfister DG, Spencer S, Adelstein D, Adkins D*, et al.* NCCN Guidelines Insights: Head and Neck Cancers, Version 1.2018. *Journal of the National Comprehensive Cancer Network : JNCCN* 2018, **16**(5)**:** 479-490.

3. ICRU report. Vol. 62: Prescribing, recording, and reporting photon beam therapy. Maryland: International Commission on Radiation Units and Measurements. 1999.

4. ICRU Report. Vol. 83: Prescribing, Recording, and Reporting Photon-Beam Intensity-Modulated Radiation Therapy (IMRT). Maryland: International Commission on Radiation Units and Measurements. 2010.

5. Sun Y, Li WF, Chen NY, Zhang N, Hu GQ, Xie FY*, et al.* Induction chemotherapy plus concurrent chemoradiotherapy versus concurrent chemoradiotherapy alone in locoregionally advanced nasopharyngeal carcinoma: a phase 3, multicentre, randomised controlled trial. *The Lancet Oncology* 2016, **17**(11)**:** 1509-1520.

6. Cao SM, Yang Q, Guo L, Mai HQ, Mo HY, Cao KJ*, et al.* Neoadjuvant chemotherapy followed by concurrent chemoradiotherapy versus concurrent chemoradiotherapy alone in locoregionally advanced nasopharyngeal carcinoma: A phase III multicentre randomised controlled trial. *European journal of cancer* 2017, **75:** 14-23.

7. Shao JY, Li YH, Gao HY, Wu QL, Cui NJ, Zhang L*, et al.* Comparison of plasma Epstein-Barr virus (EBV) DNA levels and serum EBV immunoglobulin A/virus capsid antigen antibody titers in patients with nasopharyngeal carcinoma. *Cancer* 2004, **100**(6)**:** 1162-1170.

8. Putter H, van Houwelingen HC. Understanding Landmarking and Its Relation with Time-Dependent Cox Regression. *Statistics in biosciences* 2017, **9**(2)**:** 489-503.
